# Reinduction of Bevacizumab in Combination with Pegylated Liposomal Doxorubicin in a Patient with Recurrent Glioblastoma Multiforme Who Progressed on Bevacizumab/Irinotecan

**DOI:** 10.1155/2008/942618

**Published:** 2008-09-02

**Authors:** Mohammed Almubarak, Michael Newton, Ramin Altaha

**Affiliations:** ^1^Section of Hematology/Oncology, Mary Babb Randolph Cancer Center, West Virginia University Hospital, Morgantown, WV 26506, USA; ^2^School of Pharmacy, West Virginia University, Morgantown, WV 26506, USA

## Abstract

Glioblastoma multiforme (GBM) carries a dismal prognosis despite the current standard of multimodality treatments. Recent studies showed promising results to a regimen consisting of a VEGF inhibitor, (bevacizumab) and a topoisomerase I inhibitor (irinotecan) [BI] in recurrent GBM. However, those patients with GBM who progress on BI will succumb to their disease generally in a very short period of time. We report a case of a 56-year-old male patient with GBM who declined surgical resection and received chemoradiation with temozolomide. This treatment was withheld secondary to significant thrombocytopenia. Subsequently, he achieved stable disease for 10 months with a regimen consisting of thalidomide and tamoxifen before progressing. This was followed by bevacizumab with irinotecan [BI], for which he had a significant partial response for 8 months with subsequent progression. Reinducing the patient with bevacizumab in combination with a pegylated liposomal doxorubicin [PLD] (a topoisomerase II inhibitor) demonstrated antitumor activity with significant shrinkage of contrast enhancing mass and peritumoral edema.

## 1. Introduction

Glioblastoma multiforme (GBM) is the most common primary brain tumor in adults. Despite advancement in areas of clinical detection and treatment, GBM continues to have an ominous prognosis with median survival less than a year [1]. Historically, GBM was treated with resection followed by radiation. The addition of nitrosourea-based chemotherapy added a small survival benefit. The standard of treatment was changed after Stupp et al. [2] demonstrated an improvement in survival by 2 months using temozolomide in combination with radiation compared to radiation alone.

Bevacizumab is a recombinant humanized monoclonal antibody that inhibits the biologic activity of vascular endothelial growth factor (VEGF). GBM is a highly vascularized tumor with a high expression of VEGF [3]. Bevacizumab has shown promising results in a recent phase II clinical trial when combined with a topoisomerase I inhibitor, irinotecan, for the treatment of recurrent GBM [4]. The antitumor activity of bevacizumab containing regimens could be due to enhanced permeability of the tumor vasculature which leads to improved penetration of the cytotoxic chemotherapy agents. We hypothesized that replacing irinotecan with another cytotoxic chemotherapy agent with a differenet resistance profile may overcome the resistance to BI regimen.

PLD is a liposome-encapsulated dosage form of doxorubicin which works by intercalating into DNA resulting in inhibition of DNA synthesis. It also inhibits topoisomerase II [5]. PLD has several potential advantages over standard doxorubicin including longer circulatory half-life, more specific delivery to tumor tissue and reduced cardiac toxicity [6]. A cross-resistance between irinotecan and PLD to our knowledge has not been described. Combining PLD with bevacizumab might prove to be effective in treating GBM.

## 2. Case Report

A 56-year-old healthy man presented in October 2004 with short-term memory deficit and a left-sided visual field defect. A magnetic resonance image (MRI) of the brain showed a large (5.7 cm × 3.5 cm) right occipitoparietal enhancing mass with extension into the ventricular system and satellite metastases with associated edema and mass effect. He underwent a stereotactic biopsy of the lesion that revealed a diagnosis of GBM. The patient declined surgical resection. He received steroids and was placed on levetiracetam (Keppra, UCB Inc., Smyrna, Ga, USA) for seizure prophylaxis.

**Figure 1:**
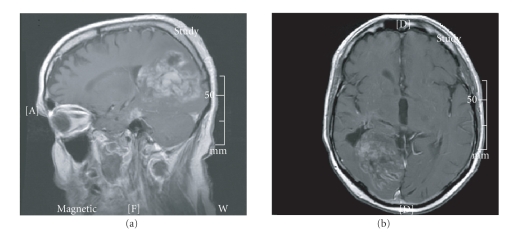
MRI brain T1 with gadolinium after stopping bevacizumab with irinotecan.

The patient was started on concomitant chemoradiotherapy per Stupp regimen consisting of temozolomide (Temodar, Schering Corporation, Kenilworth, NJ, USA) 75 mg/m^2^ daily as well as radiation therapy (a total of 59.4 Gy). He had significant improvement in his neurological function with evidence of partial response on brain MRI. However, temozolomide had to be discontinued after 2 months secondary to severe thrombocytopenia. After completion of the radiation therapy, he was then started on thalidomide 300 mg and tamoxifen 200 mg daily for maintenance. His disease was stable on this regimen for approximately 10 months. Subsequently, a brain MRI showed progression of disease. This regimen was discontinued and he was started on bevacizumab (Avastin, Genentech Inc., South San Francisco, Calif, USA) 10 mg/kg with irinotecan 125 mg/m^2^ every 2 weeks based on an abstract published by Stark-Vance in 2005 [7]. He had a 90 percent decrease in the tumor size after two cycles on this regimen. However, after seven cycles on this regimen a brain MRI showed increased mass effect and interval development of abnormal contrast enhancement in the medial hemispheric aspect of the right occipital and parietal lobe area, compatible with progression of disease (Figure 1).

**Figure 2:**
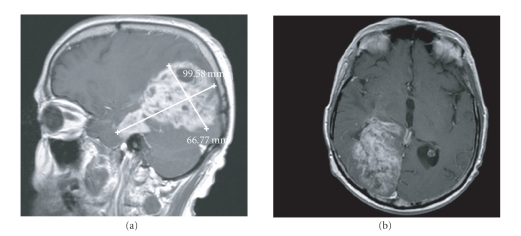
Brain T1 with gadolinium prior to reinduction with bevacizumab with PLD.

Subsequently, he was restarted on temozolomide 150 mg/m^2^ on days 1–5 every month. He developed neutropenia requiring growth factor support as well as thrombocytopenia requiring oprelvekin (Neumega, Wyeth Pharmaceuticals Inc., Philadelphia , Pa). After 2 cycles on this regimen, his follow-up brain MRI showed progression of disease (Figure 2). He was not considered a candidate for additional alkylating agent-based chemotherapy such as carmustine (BCNU) or the PCV regimen (procarbazine, lomustine, vincristine) due to dose limiting hematologic toxicity experienced with temozolomide.

**Figure 3:**
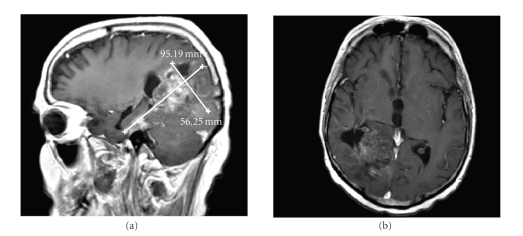
Brain T1 with gadolinium after treatment with bevacizumab with PLD.

The patient was reinduced with bevacizumab 10 mg/kg with the addition of pegylated liposomal doxorubicin (Doxil, Ortho Biotech Products, LP, Bridgewater, NJ, USA) 20 mg/m^2^ every 2 weeks. His brain MRI (T1 with gadolinium, FLAIR and T2 weighted images) after 4 treatments showed substantial decrease in bulk of the large mass in the right posterior cerebral hemisphere with associated decrease in edema (Figure 3).

Despite achieving the dramatic imaging response and initial improvement in performance status, the patient declined further chemotherapy and imaging modalities. He developed decubitus ulcer and died 2 months after he received his last dose of PLD/bevacizumab.

Table 1 summarizes the treatment regimens and duration of response for this patient.

## 3. Discussion

GBM is a highly vascularized tumor and thus targeting angiogenesis could represent a major new treatment approach. Ahmed et al. demonstrated 64% (or 9 out of 14 patients) response/disease stabilization lasting anywhere from 8 to 135 weeks using an outpatient oral regimen of the angiogenesis inhibitor, thalidomide, plus tamoxifen, and temozolomide [8].

VEGF inhibitors are currently being investigated in several clinical trials. An abstract published by Stark-Vance at the sixth meeting of the European Association of Neuro-oncology showed 43% response (9 out of 21 patients) to a regimen consisting of bevacizumab and irinotecan every 2 weeks [7]. Vredenburgh et al. further confirmed the efficacy of irinotecan and bevacizumab in patients with recurrent grade III-IV gliomas. In this phase II trial, 57% out of the 35 patients had radiographic response and the 6-month overall survival was 77%. Of note, four patients developed thomboembolic complications and one had a CNS bleed [4]. Based on this data, the National Cancer Comprehensive Network (NCCN) has updated their guidelines recently to include bevacizumab-based regimens as a salvage treatment option in recurrent GBM [9]. 

Since VEGF inhibitors affect the vascular permeability of the tumor, this may result in decrease in contrast enhancement in MRI images [10]. This might lead to questioning the true validity of using MRI to assess response to treatment. Pope et al. evaluated MRI response in high-grade gliomas treated with bevacizumab in addition to different chemotherapeutic agents. They noted reduction in contrast enhancing tumor in 7 out of 14 patients in as little as 2 weeks. Of interest, even in those patients who did not have reduction in tumors size, a reduction in edema was noted [3].

Nonetheless, response to such bevacizumab containing regimen in high-grade gliomas was proved using positron emission tomography (PET) by Chen et al. In this study, a 47% response rate (9 out of 19) was reported with 65% 6-month survival. Metabolic responders survived three times as long as nonresponders (10.8 versus 3.4 months). This was the first study to describe the ability to use PET scan as an imaging modality for predicting survival in patients who were treated with bevacizumab [11]. 

Irinotecan is a water soluble camptothecin (CPT) analog that interferes with the catalytic cycle of topoisomerase I enzyme. Irinotecan as a single agent has very low response rate in GBM [12]. The significant response noted when combined with bevacizumab could be due to improved drug delivery resulting from a reduction in interstitial fluid pressure and normalization of the tumor vasculature by bevacizumab [13]. This introduces the possibility of enhanced blood brain barrier (BBB) penetration of other cytotoxic agents when combined with bevacizumab.

Doxorubicin has shown significant in vitro cytotoxicity in cell lines derived from malignant glioma cells [14]. Unfortunately, doxorubicin does not adequately penetrate the BBB [15] and is subject to resistance due to P-glycoprotein mediated efflux [16, 17]. However, pegylated liposomal doxorubicin selectively overcomes the BBB in the tumor areas, with accumulation more than 10 fold higher in the tumor than in normal brain tissue [18]. Pegylated liposomal doxorubicin also appears to overcome P-glycoprotein mediated resistance [19]. Moreover, there seems to be an enhanced drug exposure and improvement in therapeutic activity when comparing pegylated liposomal doxorubicin to conventional doxorubicin in brain tumors in rats [20].

A single agent regimen with pegylated liposomal doxorubicin has proven moderate efficacy in GBM patients as demonstrated by Fabel et al. who observed disease stabilization in 7 of 13 patients (54%) treated with PLD 20 mg/m^2^ every 2 weeks [21]. Similarly, Hau et al. observed 40% (out of 20 patients) overall response rate and 15% six months progression-free survival using PLD 20 mg/m^2^ every 2 weeks, either alone or with tamoxifen [22]. 

In summary, previous studies demonstrated that pegylated liposomal doxorubicin penetrates BBB and achieves high-level concentration in tumor tissue. The recently described response to bevacizumab containing regimens opens the door for combining bevacizumab with other cytotoxic chemotherapeutic agents. The addition of pegylated liposomal doxorubicin to bevacizumab seems to be a reasonable alternative in recurrent GBM, as illustrated by the dramatic response observed in our patient despite heavy pretreatment. Further studies are needed to evaluate this regimen as a therapeutic option for treatment of recurrent or refractory GBM.

## Figures and Tables

**Table 1:** Summary of treatment regimens and duration of response.

Chemotherapy/treatment	Response
(1) Temozolomide and radiation	Partial response after two months, but discontinued secondary to significant thrombocytopenia.
(2) Thalidomide and tamoxifen	Disease stabilization for 10 months with subsequent progression.
(3) Bevacizumab with irinotecan	Significant partial response for 8 months with subsequent progression.
(4) Temozolomide reinduction	Disease progression after 2 months.
(5) Bevacizumab reinduction and pegylated liposomal doxorubicin	Partial response (approximately 60%) after 2 months. Treatment stopped at the patient's request.
